# Mutational and Environmental Effects on the Dynamic Conformational Distributions of Lys48-Linked Ubiquitin Chains

**DOI:** 10.3390/ijms24076075

**Published:** 2023-03-23

**Authors:** Methanee Hiranyakorn, Maho Yagi-Utsumi, Saeko Yanaka, Naoya Ohtsuka, Norie Momiyama, Tadashi Satoh, Koichi Kato

**Affiliations:** 1 Department of Functional Molecular Science, School of Physical Science, The Graduate University for Advanced Studies, SOKENDAI, 5-1 Higashiyama, Myodaiji, Okazaki 444-8787, Japan; 2 Institute for Molecular Science (IMS), National Institutes of Natural Sciences, 5-1 Higashiyama, Myodaiji, Okazaki 444-8787, Japan; 3 Exploratory Research Center on Life and Living Systems (ExCELLS), National Institutes of Natural Sciences, 5-1 Higashiyama, Myodaiji, Okazaki 444-8787, Japan; 4 Graduate School of Pharmaceutical Sciences, Nagoya City University, 3-1 Tanabe-dori, Mizuho-ku, Nagoya 467-8603, Japan

**Keywords:** allosteric effect, Lys48-linked ubiquitin chains, multidomain protein, NMR

## Abstract

In multidomain proteins, individual domains connected by flexible linkers are dynamically rearranged upon ligand binding and sensing changes in environmental factors, such as pH and temperature. Here, we characterize dynamic domain rearrangements of Lys48-linked ubiquitin (Ub) chains as models of multidomain proteins in which molecular surfaces mediating intermolecular interactions are involved in intramolecular domain–domain interactions. Using NMR and other biophysical techniques, we characterized dynamic conformational interconversions of diUb between open and closed states regarding solvent exposure of the hydrophobic surfaces of each Ub unit, which serve as binding sites for various Ub-interacting proteins. We found that the hydrophobic Ub-Ub interaction in diUb was reinforced by cysteine substitution of Lys48 of the distal Ub unit because of interaction between the cysteinyl thiol group and the C-terminal segment of the proximal Ub unit. In contrast, the replacement of the isopeptide linker with an artificial ethylenamine linker minimally affected the conformational distributions. Furthermore, we demonstrated that the mutational modification allosterically impacted the exposure of the most distal Ub unit in triUb. Thus, the conformational interconversion of Ub chains offers a unique design framework in Ub-based protein engineering not only for developing biosensing probes but also for allowing new opportunities for the allosteric regulation of multidomain proteins.

## 1. Introduction

Many proteins are constituted from multiple domains, which coordinately act to enhance affinity and specificity in molecular recognition, to form molecular networks, and to exert elaborate molecular functions, such as allosteric regulation [1]. These versatile functions of multidomain proteins involve spatial rearrangement of the domains to varying degrees [1,2]. In particular, when domains are connected by flexible linkers, which is often the case in multidomain proteins, the positions and orientations of individual domains are dynamically rearranged in wide ranges of spatial and temporal scales [3,4]. The domain rearrangements can be induced not only by ligand binding but also upon sensing changes in environmental factors, such as pH and temperature.

The features of multidomain proteins outlined above are being actively applied in the molecular design of protein engineering as best exemplified by antibody-derived multifunctional proteins [5,6]. The major challenge in such schemes is to build into the design a mechanism for controlling the coordination among domains rather than having a mere mishmash of functions in each domain. Taking the allosteric mechanism as an example, this problem can be reduced to how to link intramolecular interdomain interactions to the control of intermolecular interactions. However, solving this problem is difficult as long as we are dealing with complex multidomain proteins. We attempted to resolve this issue by dealing with simple model systems, i.e., ubiquitin (Ub) chains, which are deemed multidomain proteins with dynamic modular structures.

Ub is a small protein comprising 76 amino acid residues, which can be enzymatically connected to various proteins, including other Ub molecules, through (iso)peptide linkages, giving rise to biological messages, so-called “Ub codes”, which are read out by specific Ub-interacting proteins [7,8]. The Lys48-linked Ub chains serve as tags for protein degradation by the 26S proteasome and interact with the proteasomal subunits and other related proteins through the hydrophobic surfaces of each Ub unit [9].

According to crystallographic studies, the Lys48-linked di-Ub chain, one of the simplest units of Ub chains as multidomain proteins, often exhibits closed conformations, in which the hydrophobic surfaces are shielded through Ub-Ub interactions in chains [10,11]. In contrast, our prior NMR studies revealed that, in the major conformational state of Lys48-linked di-Ub (hereafter simply referred to as diUb), the hydrophobic surfaces are exposed to the solvent and open for the interactions with Ub-interacting proteins [12]. Furthermore, it was revealed that this two-domain protein is under dynamic equilibrium between the open and closed states depending on solution pH. Thus, in this system, the intramolecular domain–domain interaction competes with the intermolecular interactions with binding partners, suggesting that Lys48-linked Ub chains may present unique design frameworks for creating artificial multidomain proteins with dynamic structural transformation. From these viewpoints, here we evaluated the effects of modifications of environmental factors and molecular structure on the conformational distribution of Lys48-linked Ub chains.

## 2. Results and Discussion

### 2.1. Factors Affecting Intramolecular Ub-Ub Interaction

We have developed an NMR technique to quantitatively determine the conformational distributions of Ub chains [12,13]. In this approach, we use monomeric Ub (monoUb) and a cyclic form of Lys48 di-Ub (c-diUb), in which the two Ub units are connected through two isopeptide linkages, as imitations of open and closed states regarding exposure of the hydrophobic surface. NMR peaks from residues located on the hydrophobic surface, as exemplified by Val70, of each Ub unit in diUb, triUb, and tetraUb chains are aligned on the same straight line linking the corresponding peaks from monoUb and c-diUb, implying that the Ub units undergo interchange between the open and closed states in the fast exchange regime. Based on the internal division of chemical shift values, the proportions of each state can be acquired for each Ub unit (Figure S1).

**Figure 1:**
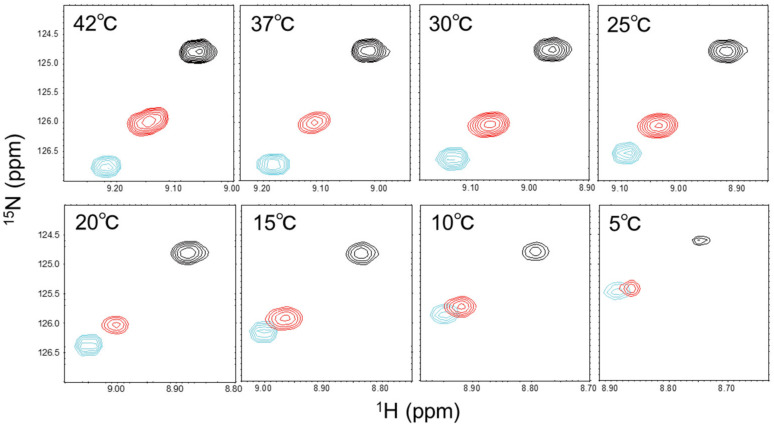
Temperature-dependent ^1^H-^15^N HSQC spectral change of diUb. ^1^H-^15^N HSQC peaks originating from Val70 of monoUb (cyan), diUb (red), and c-diUb (black) at the following different temperature conditions: 42 °C, 37 °C, 30 °C, 25 °C, 20 °C, 15 °C, 10 °C, and 5 °C.

Figure 1 depicts temperature-dependent ^1^H-^15^N HSQC spectral change of ^15^N-labeled diUb (Figure S2) along with those of monoUb and c-diUb, highlighting the application of this approach. At each temperature, the Val70 peak from diUb appeared between the corresponding peaks from monoUb and c-diUb and approached the peak from c-diUb as the temperature was raised, signifying that the proportion of the closed state increased probably due to enhanced hydrophobic interaction between the two Ub units at a higher temperature.

**Figure 2:**
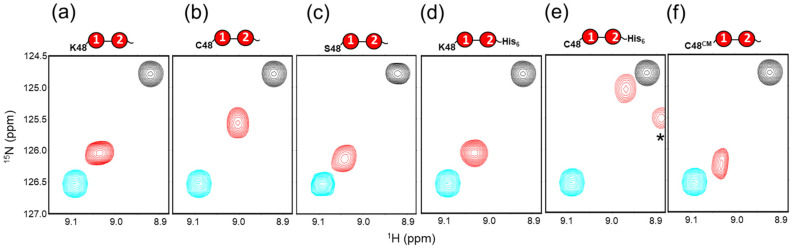
Effects of substitution of Lys48 of the distal Ub unit of diUb. ^1^H-^15^N HSQC peaks originating from Val70 of uniformly ^15^N-labeled (**a**) wild-type diUb, (**b**) K48C-diUb, (**c**) K48S-diUb, (**d**) wild-type diUb-His_6_, (**e**) K48C-diUb-His_6_, and (**f**) K48C^CM^-diUb are shown in red. The peaks from monomeric Ub and c-diUb are shown in cyan and black, respectively. The spectra were measured at 25 °C in 10 mM sodium phosphate buffer, pH 7.0. Asterisk indicates a peak originating from Phe45 of the distal Ub unit.

Besides temperature, solution pH is known to affect the proportion of open and closed states of diUb [12]. The proportion of the open state increases with decreasing pH and is overwhelmingly high at pH 4.5, indicating that the protonation of titratable groups destabilizes the closed conformation. The hydrophobic surface of Ub is surrounded by basic amino acid residues, including Lys6, Arg42, Lys48, His68, and Arg72. A previously reported mutational study implied that the ionization of the His68 side chain contributes to this destabilization by the positive repulsive charges at the Ub-Ub interface [12]. The research also implied that the positive charge of Lys48 might impact the open–closed conformational equilibrium, although this lysine residue was frequently replaced for the structural and functional characterization of Ub chains [14,15,16,17,18,19,20]. Thus, we evaluated the possible effects of the substitution of Lys48 of the distal Ub unit on the proportions of open and closed states of diUb (Figure 2).

We chose cysteine as a replacement for the Lys48 residue following the previous studies [16,17,20]. We validated that this substitution on monoUb did not influence the chemical shifts of residues on the hydrophobic surface, such as Val70 (Figure S3). Therefore, we applied the method for conformational characterization to the Lys48-mutated diUb analogs using the Val70 peaks as spectroscopic probes. The HSQC spectral data showed that the Val70 peak position of K48C-diUb was quite close to that of c-diUb, implying that this replacement substantially increased the proportion of closed states (56% at 25 °C, pH 7.0) (Figure 2b). This implies that the positive charge of Lys48 of the distal Ub unit destabilizes the closed conformation. However, serine substitution of this lysine had little impact on the proportions of closed states (75% at 25 °C, pH 7.0) (Figure 2c), signifying that, besides the elimination of electrostatic repulsion, the thiol group in K48C-diUb is a stabilizing factor for the closed conformation. The populations of open and closed forms of wild-type and mutated diUb are summarized in Table 1.

**Figure 3:**
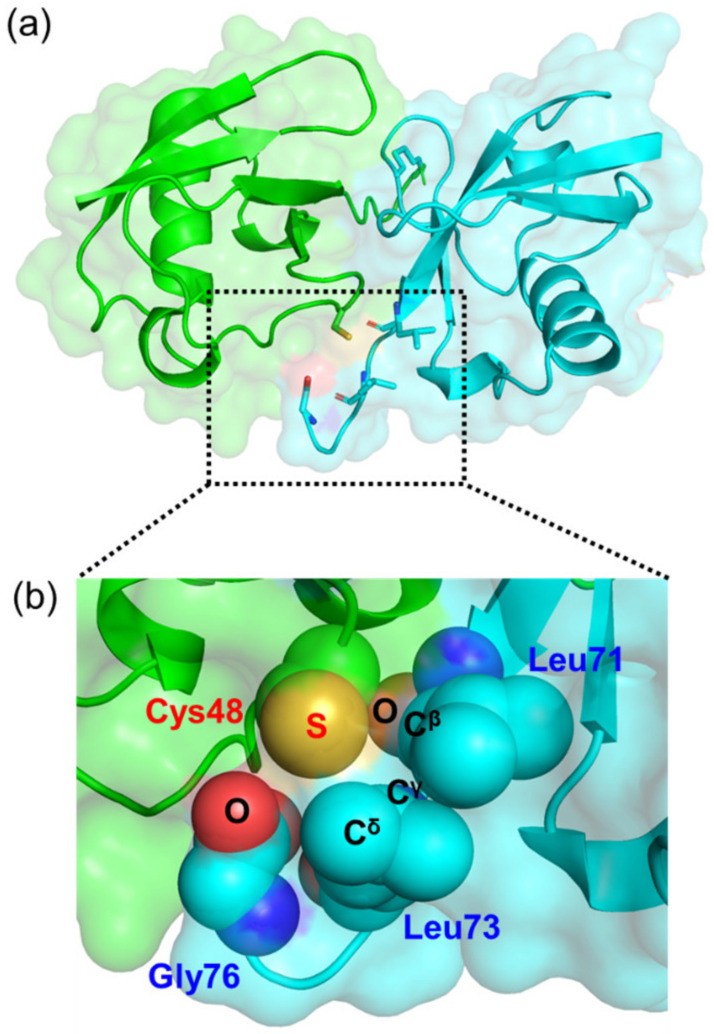
The thiol group in K48C-diUb is a stabilizing factor for its closed conformation. (**a**) Crystal structure of K48C-diUb (present structure; PDB code: 8IC9). Distal and proximal Ub units are shown in green and cyan, respectively. (**b**) The thiol group on Cys48 in the distal unit and its neighboring amino acid residues (Leu71, Leu73, and Gly76) in the proximal unit are shown in the space-filling model.

We identified the crystal structure of K48C-diUb, which closely resembled the previously reported NMR structure of c-diUb (Figure 3 and Figure S4). This crystal structure exhibits the closed conformation, the major conformation of K48C-diUb in solution. In this crystal structure, the thiol group of Cys48 of the distal Ub unit is in spatial proximity to the residues from the proximal Ub unit, including Leu71, Leu73, and Gly76, implying that atomic contacts of the thiol group with the C-terminal segment of the proximal Ub unit stabilizes the closed conformation.

The increased proportion of the closed state of K48C-diUb was further elevated by C-terminal extension of the proximal Ub with a hexahistidine tag but was counteracted by carboxymethylation (CM) of the substituting cysteine, i.e., Cys48 of the distal Ub unit (K48C^CM^-diUb), although the C-terminal extension did not impact the proportion of the closed state in wild-type diUb (Figure 2d–f). These data indicate that subtle local perturbations greatly affect the overall domain arrangement of diUb.

### 2.2. Possible Effects of Artificial Linking of Ub Units

**Figure 4:**
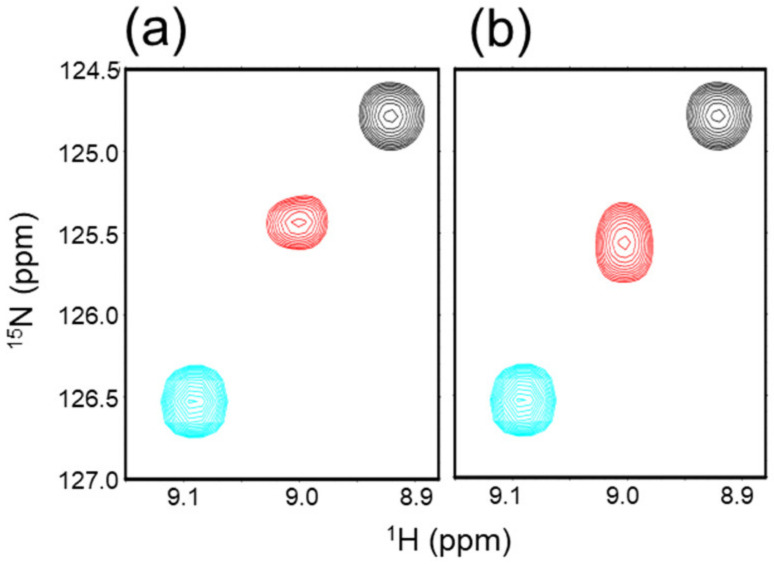
Possible effects of artificial linking of Ub units. ^1^H-^15^N HSQC peaks originating from Val70 of uniformly ^15^N-labeled (**a**) K48C-diUb^EA^ (red) and (**b**) K48C-diUb (red). The peaks from monomeric Ub and c-diUb are shown in cyan and black, respectively. The spectra were measured at 25 °C in 10 mM sodium phosphate buffer, pH 7.0.

We then investigated the potential effect of an artificial modification of the linkage between the Ub units in diUb. We applied ethylamine (EA) for chemical ligation of the two Ub units following the prior studies [20]. Two K48C-mutated Ub units were connected by the artificial EA linker (K48C-diUb^EA^). Interestingly, NMR data implied that this artificial linkage had little impact on the open–closed conformational distribution in comparison with the wild-type diUb (Figure 4, Table 1).

**Figure 5:**
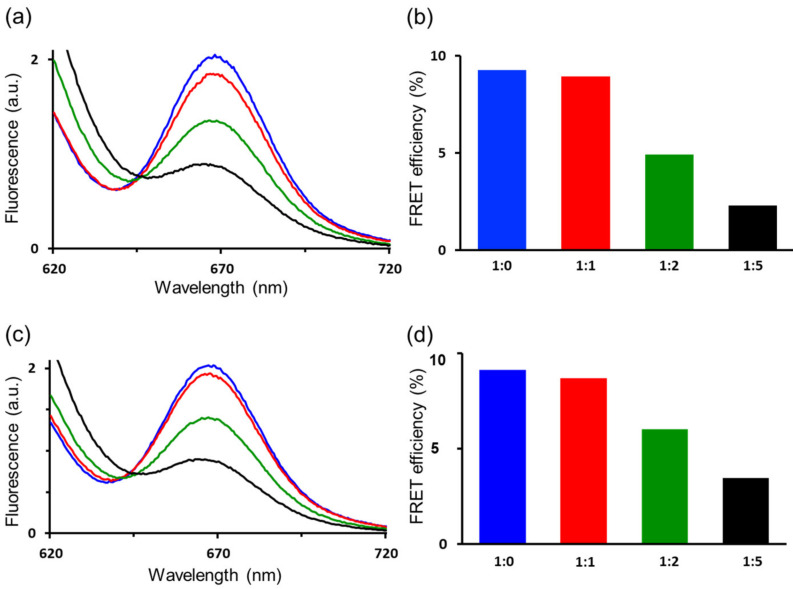
FRET verification of the possible effects of artificial linking of Ub units. The emission spectra corresponding to FRET of (**a**) Alexa647-diUb-Cy3 and (**c**) Alexa647-diUb^EA^-Cy3. The FRET efficiency calculated from the corresponding FRET intensities of (**b**) Alexa647-diUb-Cy3 and (**d**) Alexa647-diUb^EA^-Cy3. The molar ratios of diUb derivatives to E2-25K were 1:0 (black), 1:1 (red), 1:2 (green), and 1:5 (black).

We also evaluated whether the linker modification affected the dynamic conformational interchange of diUb using Förster resonance energy transfer (FRET). In this research, we employed K48C-diUb-His_6_ as a scaffold protein because its conformational equilibrium is substantially biased toward the closed form. The donor dye, Cy3 Bis NTA-Ni complex, was appended to the hexahistidine tag via a coordination bond, while the acceptor dye, Alexa Fluor 647 maleimide, was disulfide-linked to Cys48. As a result of fluorescence measurement, FRET was observed in this K48C-diUb-His_6_-based probe, Alexa647-diUb-Cy3 (Figure 5a). The FRET efficiency was not influenced by the substitution of the native isopeptide linker with the artificial one (Figure 5c).

The hydrophobic surfaces mediating the Ub-Ub interaction are physiologically involved in the interactions with various proteins in cells [7,9,21]. Therefore, Ub-interacting proteins interfere with the intramolecular Ub-Ub interaction in the closed state, reducing FRET. Indeed, upon titration with E2-25K, a Ub-conjugating enzyme harboring a Ub-binding domain, the FRET peak intensity was gradually attenuated, reflecting the closed-to-open conformational interconversion of the diUb part (Figure 5a–d). The degrees of FRET attenuation conformed between the Alexa647-diUb-Cy3 analogues with the native and the artificial linkers, indicating that the linker modification had minimal impact on the dynamic domain arrangement of diUb. FRET data also implied that the artificial linker was much less prone to deubiquitinating enzymes (DUBs) than the native isopeptide linkage, as illustrated by impaired cleavage by OTUB1, a Lys48-linkage specific DUB (Figure S5).

### 2.3. Allosteric Propagation through the Ub Chain

As demonstrated above, the intramolecular Ub-Ub interaction is mutationally controllable, providing a molecular framework for designing multidomain proteins with allosteric mechanisms. In triUb, the hydrophobic surface of the central Ub unit (Ub2) is competitively shared by the most distal and most proximal Ub units (Ub1 and Ub3, respectively). Our prior NMR analysis indicated that, among the three Ub units of triUb, Ub1 is the one most inclined to expose its hydrophobic surface [13]. Therefore, if one could improve the intramolecular interaction between Ub1 and Ub2 in triUb, this would facilitate the exposure of the hydrophobic surface of the excluded Ub3 unit.

**Figure 6:**
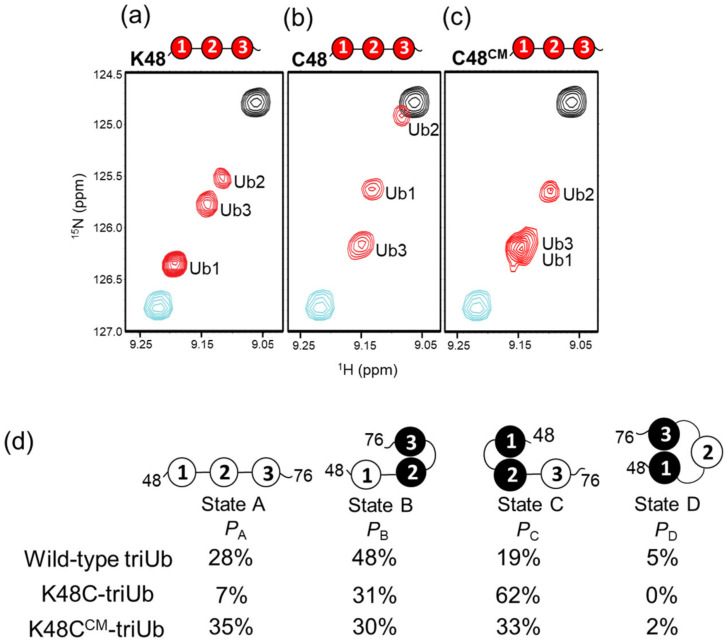
Allosteric propagation through the Ub chain. ^1^H-^15^N HSQC peaks originating from Val70 of uniformly ^15^N-labeled (**a**) wild-type triUb (red), (**b**) K48C-triUb (red), and (**c**) K48C^CM^-triUb (red). The peaks from monomeric Ub and c-diUb are plotted in cyan and black, respectively. The spectra were measured at 42 °C in 10 mM sodium phosphate buffer, pH 7.0. (**d**) Cartoon model of the conformational equilibrium of triUb. The populations of States A, B, C, and D of triUb are denoted as *P*_A_, *P*_B_, *P*_C_, and *P*_D_, respectively. The calculated *P*_A_, *P*_B_, *P*_C_, and *P*_D_ values of wild-type triUb, K48C-triUb, and K48C^CM^-triUb are indicated.

The current investigation on diUb determined that Lys-to-Cys replacement at position 48 in the distal Ub unit results in a considerable increase in the proportion of the closed state. Thus, we created the Lys48-linked triUb harboring a mutated Ub1 with the K48C substitution (K48C-triUb) and subjected it to NMR spectral measurements. As depicted in Figure 6, K48C-triUb gave three peaks from Val70 along the straight line linking the Val70 peaks from monoUb and c-diUb; however, the peak positions were significantly distinct from those of wild-type triUb. Using ^15^N-labeled K48C-triUb chains unit-selectively, we made assignments of the individual peaks, allowing quantification of the conformer distribution (Figure S6).

Our prior NMR analysis demonstrated that, in wild-type triUb, 76% of all conformers have the Ub1 hydrophobic surface exposed to solvent (States A and B): Ub1 interacted with Ub2 in only 19% of the conformers (State C) [13]. In contrast, in K48C-triUb, Ub1 interacting with Ub2 accounted for 62% of the conformers (Figure 6b,d). Consequently, the percentage of the conformational states with exposure of the Ub3 hydrophobic surface (States A and C) increased from 47% to 69%. As anticipated from the outcomes of the analysis of diUb, carboxymethylation of Cys48 of Ub1 resulted in a decrease (to 33%) in the proportion of the conformer with Ub1-Ub2 interaction (State C) and concomitant increase (to 35%) in the conformational state without the hydrophobic, surface-mediated Ub-Ub interaction (State A) in triUb (Figure 6c,d).

**Figure 7:**
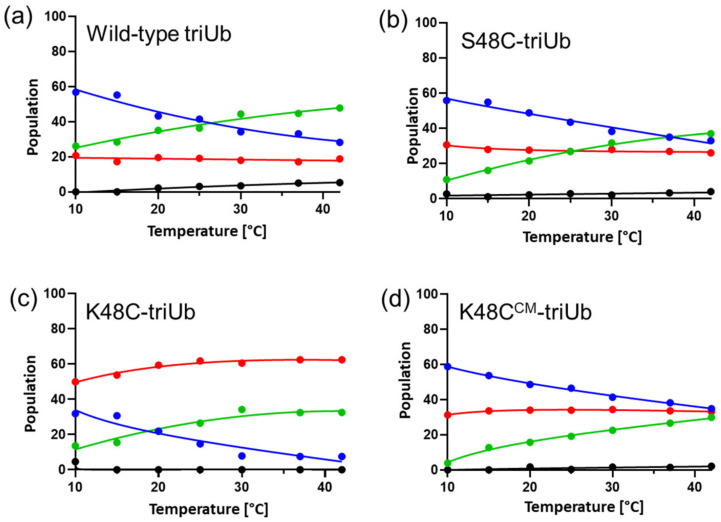
Quantitative estimation of the temperature-dependent populational shift of each conformer of triUb and its analogues. Temperature dependence of the open conformational population of (**a**) wild-type triUb, (**b**) K48S-triUb, (**c**) K48C-triUb, and (**d**) K48C^CM^-triUb. The calculated *P*_A_, *P*_B_, *P*_C_, and *P*_D_ values are shown in blue, green, red, and black, respectively. The population of each temperature was estimated based on the dividing ratio of chemical shift differences of Val70.

We also assessed the temperature dependence of the conformational equilibrium of triUb by performing NMR measurements at multiple temperatures. The Val70 peak positions altered with temperature but were still aligned in the same straight line between those from c-diUb and monoUb, allowing a quantitative calculation of the temperature-dependent populational shift of each conformer of triUb and its analogues, i.e., K48S-triUb, K48C-triUb, and K48C-carboxymethyl-triUb (K48C^CM^-triUb) (Figure 7). In all the triUb derivatives, the proportions of States A and B revealed a temperature dependency: with increasing temperature, the proportion of State B increased with a concomitant decrease in the State A proportion. The closed structure created between the Ub2 and Ub3 units in State B is supposed to be mediated through hydrophobic interactions, which are enhanced at higher temperatures, as seen in diUb. In contrast to States A and B, the population of State C was found to be constant, regardless of temperature. These data highlight that the conformational distribution of triUb is identified by the competition between the Ub1-Ub2 interaction, which depends on modification at position 48 of the Ub1 unit but not on temperature, and the Ub2-Ub3 interaction, which is governed by the endothermic hydrophobic interaction.

## 3. Materials and Methods

### 3.1. Preparation of Lys48-Linked Ub Chains

Human Ub, C-terminally hexahistidine-tagged Ub (Ub-His_6_), and K48S-mutated Ub were expressed and purified as described previously [12,13]. K48C-mutated Ub was produced using site-directed mutagenesis techniques and was purified by the same protocol employed for the wild-type Ub. To yield the isotopically labeled protein, the wild-type and mutated Ub were expressed from the pGEX6p-1 plasmid in *Escherichia coli* BL21(DE3) CodonPlus cells, which were grown in M9 minimal media containing [^15^N]NH_4_Cl (1 g/L). Ub-related enzymes, such as Ub E1, E2-25K, glutathione S-transferase-tagged Cdc34 (GST-Cdc34), and yeast ubiquitin hydrolase 1 (YUH1), were expressed and purified as described previously [13].

The cyclic and native forms of the Lys48-linked Ub chains were prepared by an in vitro enzymatic reaction using E2-25K and GST-Cdc34, respectively, as described previously [12,13]. After the reaction, the Lys48-linked Ub chains possessing the hexahistidine tag were separated using Mono S (Cytiva, MA, USA) cation exchange chromatography. To cleave the hexahistidine tag, the Ub chains were treated with YUH1. The Ub chains without the hexahistidine tag were purified by MonoS cation exchange chromatography and size exclusion chromatography using a Superdex 75 column (Cytiva, MA, USA). The subunit at the distal end of the triUb chains was termed Ub1, and the remaining subunits were numbered sequentially. The Ub chains with an amino acid substitution at position 48 of the distal Ub unit were prepared using the same protocol.

### 3.2. Carboxymethylation of K48C-diUb and K48C-triUb

For carboxymethylation of the thiol group, 1 mg/mL of K48C-diUb or K48C-triUb was incubated with 10 mM DTT in 100 mM Tris-HCl (pH 8.5) at 25 °C for 4 h. Subsequently, 25 mM iodoacetic acid was mixed into the solution, which was then incubated for 30 min in the dark. After the addition of 20 mM DTT for quenching the iodoacetic acid, carboxymethylated K48C-triUb was purified by Mono S cation exchange chromatography and size exclusion chromatography using a Superdex 75 column.

### 3.3. Preparation of K48C-diUb with an Ethylamine Linker

The EA linker was synthesized as described previously [22]. K48C-Ub-His_6_ was incubated with EA at 37 °C for 4 h for their conjugation. K48C-Ub and EA-conjugated K48C-Ub-His_6_ were mixed at a molar ratio of 2:1 in 50 mM Tris-HCl (pH 8.0) in the presence of GST-Cdc34 for the creation of EA-mediated K48C-diUb-His_6_ (K48C-diUb^EA^-His_6_). Following the reaction, K48C-diUb^EA^-His_6_ was isolated by MonoS cation exchange chromatography. To remove the hexahistidine tag, K48C-diUb^EA^-His_6_ was treated with YUH1 and then subjected to chromatographic separation, as described in Section 3.1.

### 3.4. NMR Measurements

All of the NMR samples were prepared in 10 mM sodium phosphate buffer, pH 7.0, containing 1 mM DTT in 95% H_2_O/5% D_2_O (*v*/*v*). NMR spectra were recorded at varying temperatures on Bruker AVANCE-III HD 500 or AVANCE 800US spectrometers equipped with 5 mm cryogenic triple-resonance probes. The spectral data were processed using NMR Topspin and analyzed using Sparky [23].

### 3.5. Crystallization, X-ray Data Collection, and Structure Determination

For crystallization, purified K48C-diUb was dissolved in a protein concentration of 8.0 mg/mL in 20 mM Tris-HCl (pH 8.0). Protein crystals were grown in a buffer containing 38% 2- methyl-2,4-pentanediol and 50 mM sodium citrate (pH 4.0), with incubation at 4 °C, and directly frozen by means of liquid nitrogen using the mother liquor. The diffraction data were integrated and scaled using XDS [24]. The crystal of K48C-diUb belonged to the space group *P*1 and was diffracted up to a resolution of 1.25 Å. The crystal structure of K48C-diUb was solved by the molecular replacement method using the program MOLREP [25] with a human Lys48-linked K48C-diUb (Protein Data Bank code: 1AAR) as a search model.

Model fitting to the electron density maps and the subsequent refinement were performed using COOT [26] and REFMAC5 [27], respectively. The stereochemical quality of the final model was validated using MolProbity [28]. The data collection and refinement statistics for K48C-diUb are summarized in Table 2. The molecular graphics were prepared using PyMOL (Schrödinger, NY, USA). The coordinates and structural factors for the crystal structure of the Lys48-linked K48C-diUb were deposited in the Protein Data Bank under accession number 8IC9.

### 3.6. FRET Measurements and Analyses

Alexa647-maleimide and Cy3 Bis NTA-Ni were purchased from Thermo Fisher Scientific (Waltham, MA, USA) and AAT Bioquest (Sunnyvale, CA, USA), respectively. K48C-diUb-His_6_ or K48C-diUb^EA^-His_6_ was incubated in 50 mM Tris-HCl (pH 7.2) overnight at 4 °C at a protein concentration of 80 µM in the presence of 0.8 mM Alexa647-maleimide and 10 mM TCEP. The excess amounts of unincorporated dyes and unconjugated proteins were removed by MonoS cation exchange chromatography. Alexa647-conjugated K48C-diUb-His_6_ or K48C-diUb^EA^-His_6_ at a protein concentration of 100 µM in 10 mM sodium phosphate buffer, pH 7.0, was mixed with an equimolar amount of Cy3 Bis NTA-Ni dye, giving rise to Alexa647-diUb-Cy3 or Alexa647-diUb^EA^-Cy3, respectively.

Fluorescence emission spectra were recorded with 0.8 µM Alexa647-diUb-Cy3 or Alexa647-diUb^EA^-Cy3 after 30 min incubation in 10 mM sodium phosphate buffer, pH 7.0, at 25 °C using a fluorescence spectrometer (Hitachi F2700, Japan) in the presence and absence of varying concentrations of E2-25K or OTUB1 (Life sensors, PA, USA). Emission spectra were recorded at 400–800 nm with an excitation wavelength of 550 nm. FRET peaks were observed at 670 nm with an excitation wavelength of 550 nm. FRET efficiency was calculated using FRET efficiency = (total FRET spectral − spectral bleed-through − cross-excitation)/emission of donor (Cy3). Monomeric forms of Ub-His_6_-Cy3 and K48C-Alexa647 were employed for measuring spectral bleed-through (Ex: 550 nm, Em: 570 nm) and for cross-excitation (Ex: 650 nm, Em: 670 nm), respectively. To evaluate the susceptibility of the diUb chains to DUB, 100 µM Alexa647-diUb-Cys3 and Alexa647-diUb^EA^-Cy3 were incubated with 20 µM OTUB1 at 37 °C for 1 h in the presence of 2 mM DTT. Subsequently, the solution was diluted to 0.8 µM with 10 mM sodium phosphate buffer, pH 7.0, and then 0.8 µM Cy3 Bis NTA-Ni dye was added for FRET measurements.

## 4. Conclusions

The conformational interconversion property of diUb offers a unique design framework in Ub-based protein engineering for developing biosensing probes to sense environmental conditions, such as temperature and pH, as well as to detect binding molecules. The current findings underscore that the open–closed conformational equilibrium of diUb is sensitive to modifications of position 48 of the distal Ub unit, providing an opportunity for controlling its conformational distribution. In more complicated systems comprising three or more Ub units, the hydrophobic surface of each Ub unit can be competed for not only by the binding partners but also by the other two or more Ub units. Under such circumstances, a perturbation in one Ub unit can influence the solvent exposure of the ligand binding sites of the remaining Ub units. This was demonstrated by the K48C mutation in Ub1 of triUb, which enhanced exposure of the hydrophobic surface of Ub3. Such allosteric effects can be transmitted to more distal sites in longer Ub chains in a chain-reaction manner, resulting in their functional modulation. The linker connecting Ub units can also be a design target for functional optimization. Furthermore, Ub chains can be fused to other multidomain proteins even with enzymatic cyclization by isopeptide linking [29]. Thus, Ub-based protein engineering will open new possibilities for the allosteric regulation of multidomain proteins.

## Figures and Tables

**Table 1:** The populations of open and closed forms at 25 °C, pH 7.0.

	Open (%)	Closed (%)
Wild-type diUb	71	29
K48C-diUb	44	56
K48S-diUb	75	25
Wild-type diUb-His_6_	71	29
K48C-diUb-His_6_	18	82
K48C^CM^-diUb	76	24
K48C-diUb^EA^	38	62

**Table 2:** Data collection and refinement statistics for the crystal structure of K48C-diUb.

Crystallographic Data		
Space group		*P*1
Unit cell	*a*/*b*/*c* (Å)	86.79/31.19/25.27
	α/β/γ (°)	90.00/106.93/88.65
**Data Processing Statistics**		
Beamline		SPring-8 BL44XU
Wavelength (Å)		0.9000
Resolution (Å)		19.64–1.25 (1.27–1.25)
Total/unique reflections		241,895/66,682
Completeness (%)		95.1 (93.6)
*R*_merge_ (%)		6.2 (55.4)
*I*/σ (*I*)		6.7 (2.7)
**Refinement Statistics**		
Resolution (Å)		19.64–1.25
*R*_work_/*R*_free_ (%)		18.6/21.9
RMS deviations from ideal		
Bond length (Å)		0.011
Bond angle (°)		1.57
Ramachandran plot (%)		
Favored		100
Allowed		0
Outliers		0

## Data Availability

The atomic coordinates and structure factors are deposited in the Protein Data Bank with accession code 8IC9. The data presented in this study are available on request from the corresponding author.

## Supplementary Materials

The following supporting information can be downloaded at: https://www.mdpi.com/article/10.3390/ijms24076075/s1.
