# Supplementary material for: Mutational and Environmental Effects on the Dynamic Conformational Distributions of Lys48-Linked Ubiquitin Chains
Source: Int J Mol Sci. 2023 Mar 23;24(7):6075. doi: 10.3390/ijms24076075 (PMC10094362; doi:10.3390/ijms24076075)
Supplement: Supplementary file 1 [file ijms-24-06075-s001.zip › ijms-2274615-supplementary.pdf]

Supplementary Materials

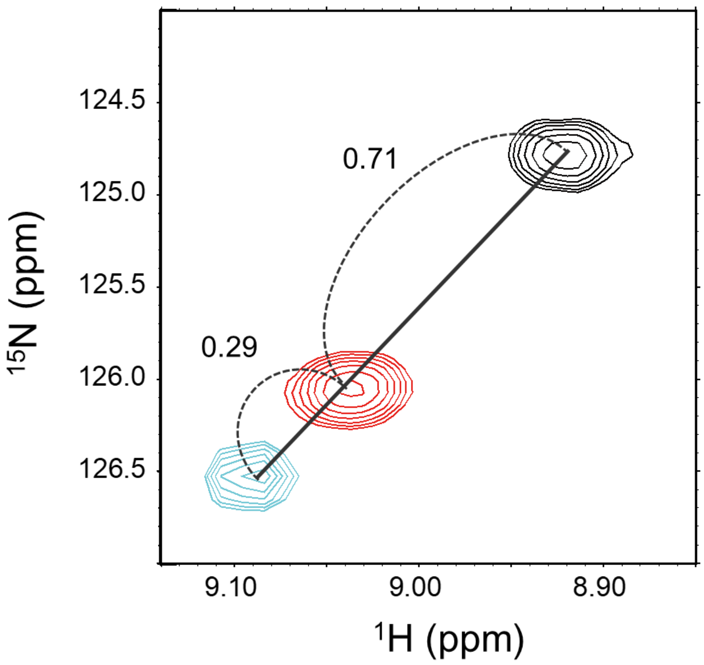

Figure S1. The population of open and closed forms of wild-type diUb. ^{1}H-^{15}N HSQC peaks originating from Val70 of monoUb (cyan), diUb (red), and c-diUb (black) at 25°C, pH 7.0. Val70 located on the hydrophobic surfaces of diUb exhibited an HSQC peak between the peaks originating from the corresponding sites in monoUb and c-diUb in the same straight line, indicating that diUb was in a conformational equilibrium between the open and closed states, which were mimicked by monomeric Ub and cyclic diUb, respectively, with regard to the exposure of the hydrophobic surfaces to the solvent. Thus, the dividing ratio of the chemical shift difference indicates the population of open and closed conformers, shown as 0.71 and 0.29, respectively.

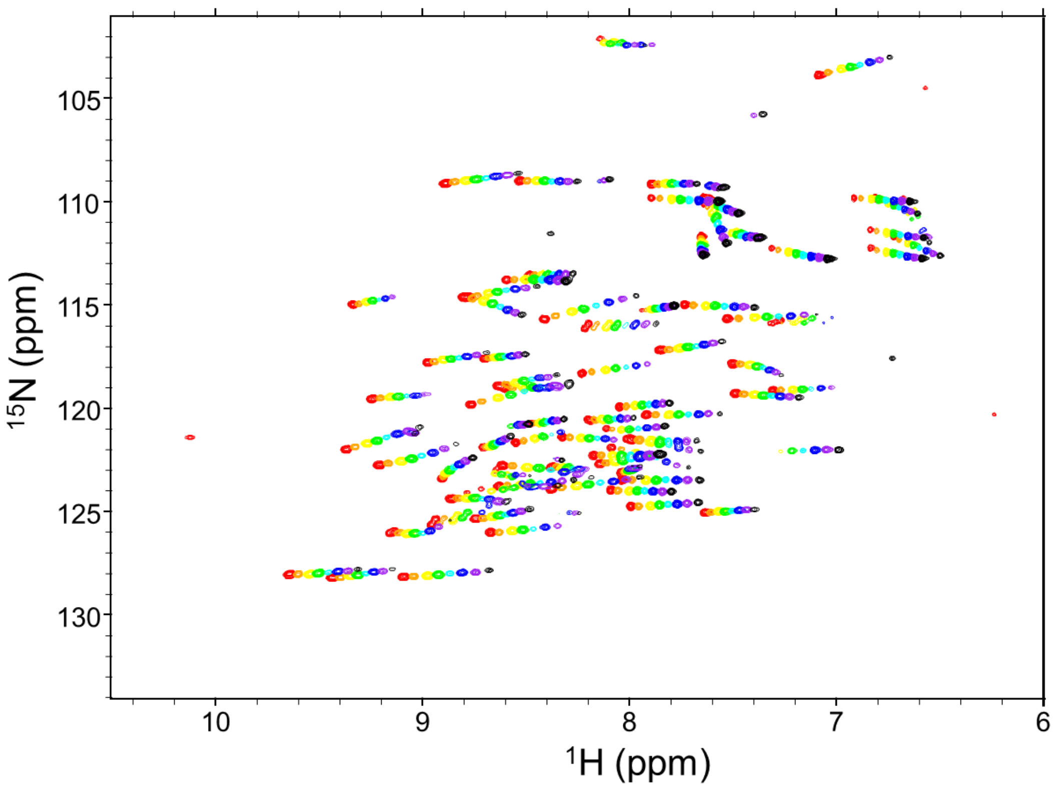

Figure S2. Temperature-dependent ^{1}H-^{15}N HSQC spectral changes of diUb. ^{1}H-^{15}N HSQC spectra of diUb measured at the following different temperature conditions: 5°C (black), 10°C (purple), 15°C (blue), 20°C (cyan), 25°C (green), 30°C (yellow), 37°C (orange), and 42°C (red).

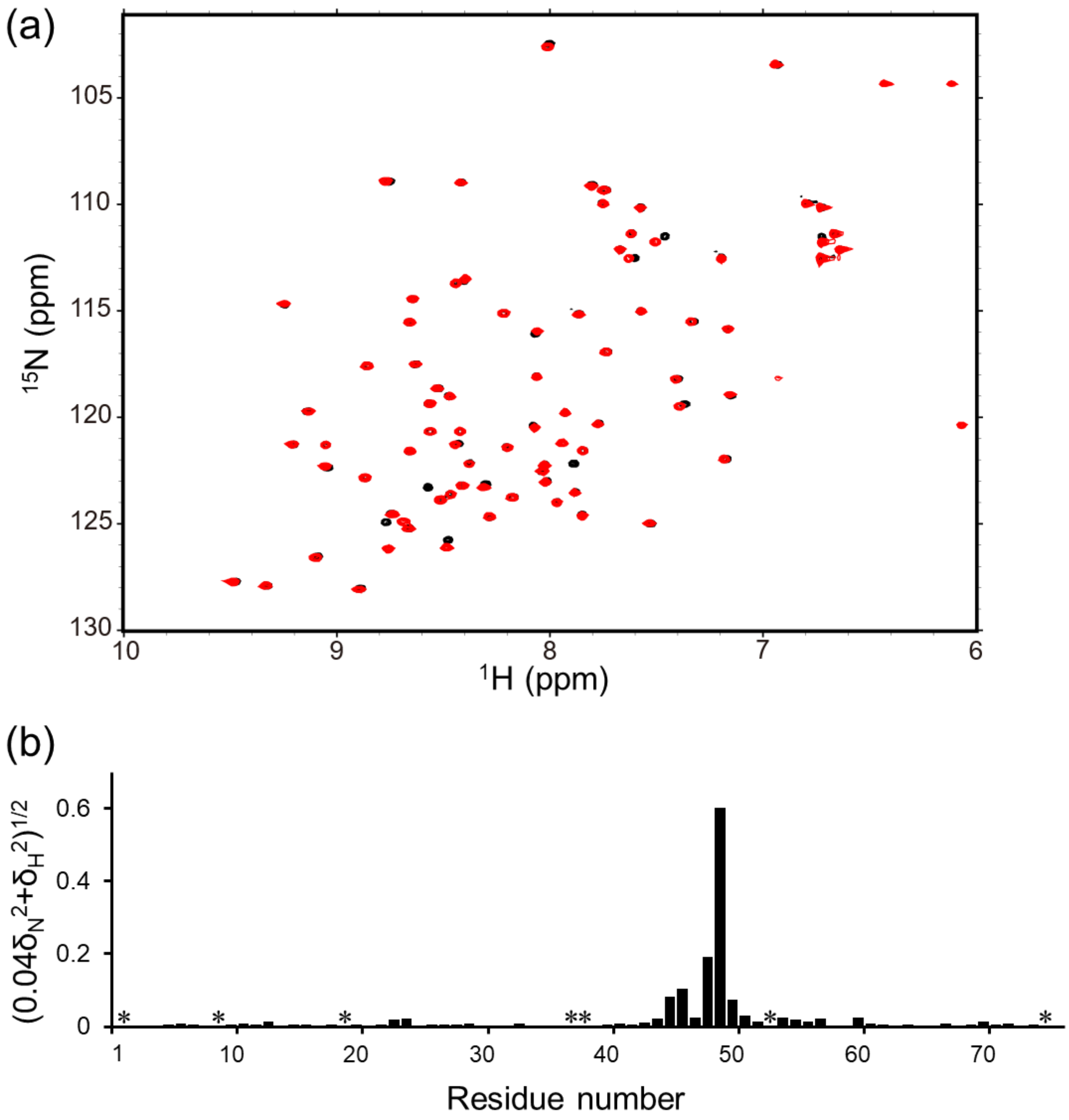

Figure S3. Spectral comparison of monomeric K48C-Ub with wild-type monoUb, (a) ^{1}H-^{15}N HSQC spectra of monomeric K48C-Ub (red) with wild-type monoUb (blue). (b) Chemical shift differences between monomeric K48C-Ub and wild-type monoUb. Data are shown according to the equation $(0.04\delta N^2 + \delta H^2)^{1/2}$, where δN and δH represent the difference in nitrogen and proton chemical shifts, respectively. The proline residues and the residues whose ^{1}H-^{15}N HSQC peak could not be used as a probe because of broadening are shown by asterisks.

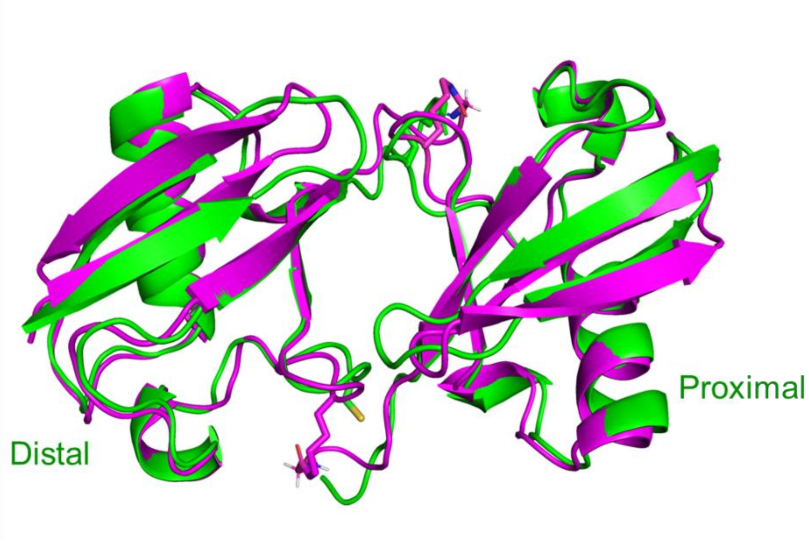

Figure S4. Both of the crystal structure of K48C-diUb (green, PDB code: 8IC9) and NMR-derived model of c-diUb [1] (magenta) show the closed conformation.

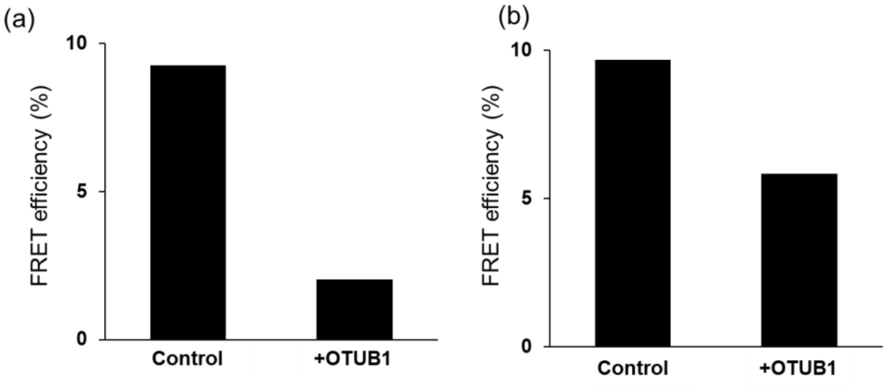

Figure S5. DUB resistance of artificial linker**.** FRET efficiency of (a) Alexa647-diUb-Cy3 and (b) Alexa647-diUb^{EA}-Cy3 calculated from the corresponding FRET intensities with and without OTUB1.

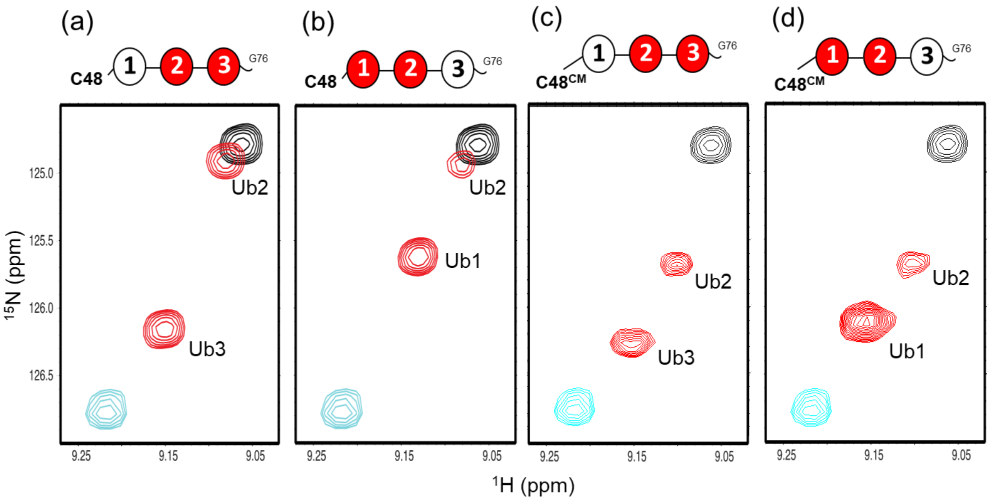

Figure S6. Spectral assignments of the individual peaks of K48C-triUb and K48C^{CM}-triUb. ^{1}H-^{15}N HSQC peaks originating from Val70 of unit-selectively ^{15}N-labeled (a) K48C-triUb at Ub2 and Ub3, (b) K48C-triUb at Ub1 and Ub2, (c) K48C^{CM}-triUb at Ub2 and Ub3, and (d) K48C^{CM}-triUb at Ub1 and Ub2. The spectra were measured at 42°C in 10 mM sodium phosphate buffer, pH 7.0.

Reference

1. Hirano, T.; Serve, O.; Yagi-Utsumi, M.; Takemoto, E.; Hiromoto, T.; Satoh, T.; Mizushima, T.; Kato, K., Conformational dynamics of wild-type Lys-48-linked diubiquitin in solution. *J Biol Chem* **2011,** 286, (43), 37496-502.
